# Hypoglycemic agents for non-alcoholic fatty liver disease with type 2 diabetes mellitus: A protocol for systematic review and network meta-analysis

**DOI:** 10.1097/MD.0000000000021568

**Published:** 2020-08-07

**Authors:** Su-Tong Liu, Kai-Qi Su, Li-Hui Zhang, Ming-Hao Liu, Wen-Xia Zhao

**Affiliations:** a Henan University of Chinese Medicine; b Department of Gastroenterology, The First Affiliated Hospital of Henan University of Chinese Medicine, Zhengzhou, Henan, PR China.

**Keywords:** hypoglycemic agents, network meta-analysis, non-alcoholic fatty liver disease, systematic review, type 2 diabetes mellitus

## Abstract

**Background:**

Non-alcoholic fatty liver disease (NAFLD) is the most common cause of chronic liver disease in Western countries, and strongly associated with type 2 diabetes mellitus (T2DM). Several studies have shown that hypoglycemic agents are effective for NAFLD combined with T2DM. However, there is still controversy over which hypoglycemic agent is the best for NAFLD combined with T2DM patients.

**Objective:**

To systematically evaluate the efficacy and safety of hypoglycemic agents in NAFLD combined with T2DM patients.

**Methods:**

A comprehensive electronic search will be conducted by searching Web of Science, PubMed, EMBASE, Cochrane Central Register of Controlled Trials, Clinical Trials and Chinese Biomedical Medicine. All randomized controlled trials of hypoglycemic agents interventions for NAFLD combined with T2DM will be identified. Two reviewers independently screened and evaluated each included study and extracted the outcome indexes. ADDIS 1.16.8 software will be used for the network meta-analysis and STATA 14 software will be used for drawing network evidence plots and funnel plots.

**Conclusion:**

This network meta-analysis will provide stronger evidence for the efficacy and safety of hypoglycemic agents in the treatment of NAFLD combined with T2DM, and provide a reference for clinical application.

**Protocol registration number:**

INPLASY202070016.

## 1 Introduction

Non-alcoholic fatty liver disease (NAFLD) is a spectrum characterized by a pathological feature of diffuse bullous steatosis of hepatocytes, may progress to non-alcoholic steatohepatitis, liver fibrosis, cirrhosis, and hepatocellular carcinoma.^[1–3]^ NAFLD has become one of the main causes of chronic liver disease in many regions of the world.^[4,5]^ According to statistics, the prevalence of NAFLD is roughly 25% to 30% in the general population in developed countries.^[6]^ Since metabolic syndrome or type 2 diabetes mellitus (T2DM) is strongly associated with NAFLD, the prevalence of NAFLD has been reported to reach 70% in patients affected by T2DM.^[7–9]^ Insulin resistance plays a key role in pathogenic mechanisms of NAFLD and it acts as a trigger for progression of steatosis towards steatohepatitis, cirrhosis, and end-stage complications.^[10,11]^ Similarly, several articles have shown that NAFLD also substantially increased the risk of T2DM.^[12–14]^ NAFLD interacts with T2DM, and insulin resistance is the main driving factor. Therefore, hypoglycemic agents have been used in the treatment of NAFLD and T2DM.^[15,16]^

At present, commonly used hypoglycemic agents can be divided into the following categories according to the different mechanisms of action: insulin and insulin analogs, biguanides, sulfonylurea secretagogues, α-glycosidase inhibitors, insulin sensitizers, dipeptidyl peptidase 4 inhibitors, sodium glucose cotransporter 2 inhibitors, and glucagon-like peptide-1 receptor agonists.^[16–18]^ In China, the prices of these different drugs vary widely. Although there have been several published studies on single drug versus placebo or single drug versus another single drug, there is still no relevant systematic study on the efficacy and safety of those various hypoglycemic agents in the treatment of NAFLD combined with T2DM.^[19–21]^ Therefore, there is no strong evidence to support which drug is appropriate for patients, the newer, the better; or the more expensive, the better? In this study, the efficacy and safety of multiple hypoglycemic agents will be compared to provide a bias for patients to selectively take hypoglycemic agents.

## 2 Methods

### 2.1 Design and registration

The systematic review protocol will be reported in accordance with the guidelines of Cochrane Handbook for Systematic Reviews of Interventions and the Preferred Reporting Items for Systematic Reviews and Meta-analysis Protocol Statement.^[22]^ The protocol has been registered on the INPLASY website (https://inplasy.com/inplasy-2020-7-0016/) and INPLASY registration number is INPLASY202070016. No ethical approval is required since this study used data already in the public domain.

### 2.2 Inclusion criteria

#### 2.2.1 Study design

Only clinical randomized controlled trials (RCTs) published or registered but not yet published in both Chinese and English using hypoglycemic agents to treat NAFLD combined with T2DM will be selected for this study. However, studies from animal experiments, reviews, case reports, and non-RCTs will be excluded.

#### 2.2.2 Participants

Participants were individuals who are clinically diagnosed with NAFLD combined with T2DM and are simultaneously treated with hypoglycemic agents. There are no restrictions on race, gender, or age. Participants with NAFLD caused by other reasons and participants with severe heart disease, liver and kidney dysfunction, mental illness, pregnant or allergies to hypoglycemic agents will be excluded.

#### 2.2.3 Interventions

Both groups of participants were treated with different hypoglycemic agents, or 1 group received hypoglycemic agents, while the other group received placebo, or no treatment was applied. In addition, neither group took any drugs that interfered with the outcome indicators.

#### 2.2.4 Outcomes

The primary outcomes include the improvement in clinical efficacy and imaging markers, biomarkers of hepatic steatosis, serological indexes of hepatic fibrosis, serum NAFLD liver fat score.

Secondary outcomes are mainly composed of fasting blood glucose, 2 hours postprandial blood glucose, HbA1c, serum insulin levels, aspartate transaminase, alanine transaminase, γ-glutamyl transferase, and adverse events.

### 2.3 Search methods

**Figure 1:**
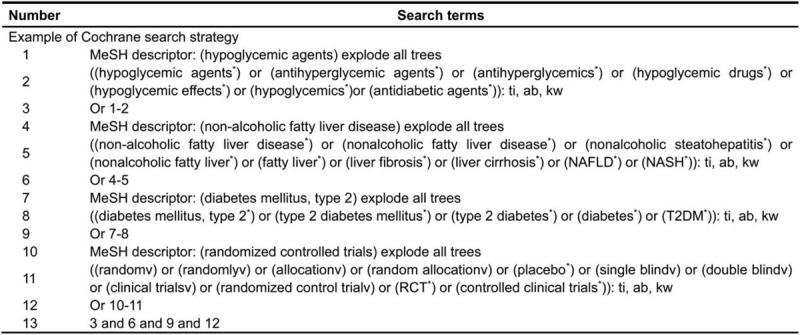
Cochrane Library search strategy.

We will search publications in the following English and Chinese database: Web of Science, PubMed, EMBASE, Cochrane Central Register of Controlled Trials, Clinical Trials and Chinese Biomedical Medicine, and the retrieval time will be set to the time of database-building to May 31, 2020. The combination of subject headings and free words will be used as a search strategy for a comprehensive electronic search. Subject headings include “hypoglycemic agents,” “non-alcoholic fatty liver disease,” “diabetes mellitus, type 2,” and “randomized controlled trials.” In Figure 1, we present the search strategy of the Cochrane Library.

### 2.4 Study screening, data extraction, and risk assessment of bias

First, all the retrieved data will be imported into Endnote X7 software. Two researchers will independently screen and remove the literature that clearly does not meet the inclusion criteria by reading the title and abstract, and eliminate the research that does not meet the requirements, and then carefully read the full text and select the research that meet the requirements. Second, we will carefully read the full text of the remaining literature to further decide whether to include or not. Finally, the 2 researchers will independently extract the data and carefully check it by a third researcher. If there are any disagreements during the data collection process, we will reach agreement through discussion or seek advice from a third party. The data we extract include the following:

(1) the basic information of the research, including the title, first author, year of publication and country;
(2) the characteristics of the research, including the number of participants in the experimental group and the control group, ethnicity, and the intervention measures and treatment duration of each group;
(3) outcomes indicators and data;
(4) information required to assess the risk of bias.

We will use the RCT bias risk assessment tool recommended by the Cochrane Handbook for Systematic Reviews of Interventions 5.1.0 to perform bias risk assessment and methodological quality assessment of included RCTs.^[23,24]^

### 2.5 Statistical analysis

In this study, ADDIS 1.16.8 software will be used to perform the network meta-analysis through the Bayesian hierarchical model. For the dichotomous variables, the odds ratio will be used as an effect indicator, while for continuous variable, the weighted mean difference or standard mean difference will be used. The results of the effect analysis statistics will be presented by the estimated value and 95% confidence interval, and we will set the significance level at α* = *0.05. Heterogeneity testing will be implemented to detect statistical heterogeneity between each study. If there is no heterogeneity, then network meta-analysis will be performed directly. If there is heterogeneity, then we will analyze and describe the source of the heterogeneity. The inconsistency test will be carried out using the node-split model. If *P* > .05, it means that there is no significant statistical difference between direct and indirect comparison, then the consistency model will be selected for analysis; otherwise, the inconsistency model will be selected.^[25,26]^ We will use a sorted probability table to rank the probabilities of each intervention, further analyze the advantage and disadvantages of each intervention. We will use the STATA 14 software to draw a network diagram showing the comparisons between them and a funnel plot can be used to make a qualitative judgment of publication deviation.

### 2.6 Subgroup analysis

If necessary, we will conduct subgroup analyses on characteristics such as gender, age, race, nationality, type of hypoglycemic agents, and duration of medication to explore whether treatment effects for our primary outcomes are robust.

### 2.7 Assessment of publication bias

We will evaluate the publication bias by making funnel plots, of course, this is under the condition that more than 15 articles can be quantitatively analyzed. If the data in the funnel plots are symmetrically distributed, it means that there is no publication bias, otherwise we will analyze the possible reasons and give a reasonable explanation for asymmetric funnel plots.^[27]^

### 2.8 Assessment of quality of evidence

We will use the Grading of Recommendations Assessment, Development, and Evaluation system to evaluate the quality of our evidence. In this system, the level of evidence quality is divided into 4 levels: high quality, moderate quality, low quality, and very low quality.^[28]^

## 3 Discussions

Studies have shown that NAFLD is a multi-system disease that can affect multiple extrahepatic organ systems and interact with multiple metabolic/endocrine and pro-inflammatory pathways.^[29,30]^ There is convincing evidence that NAFLD is closely associated with T2DM, and the simultaneous prevalence of 2 diseases is a major global challenge.^[4,31]^ However, approved treatments for NAFLD combined with T2DM are limited.

Hypoglycemic agents have been widely used in the first-line treatment of NAFLD combined with T2DM, and their efficacy and safety should be guaranteed. In addition, hypoglycemic agents may be drugs that patients are required to take for life, so it is particularly necessary to provide patients with more information on the efficacy and safety of hypoglycemic agents in the treatment of NAFLD combined with T2DM, so as to better help patients control disease progression and reduce treatment costs.

In conclusion, this study will conduct a systematic review and network meta-analysis of relevant RCTs, and will comprehensively assess whether hypoglycemic agents are beneficial to NAFLD combined with T2DM. In addition, this study will compare the advantages and disadvantages of various hypoglycemic agents, and provide higher quality evidence for the selective use of hypoglycemic agents in NAFLD with T2DM patients and guide clinical practice.

## Author contributions

**Conceptualization**: Su-Tong Liu, Kai-Qi Su.

**Data curation**: Li-Hui Zhang, Wen-Xia Zhao.

**Methodology**: Su-Tong Liu, Kai-Qi Su

**Software**: Kai-Qi Su, Ming-Hao Liu.

**Supervision**: Su-Tong Liu, Wen-Xia Zhao.

**Writing – original draft:** Su-Tong Liu, Kai-Qi Su, Li-Hui Zhang, Ming-Hao Liu, Wen-Xia Zhao.

**Writing – review & editing:** Su-Tong Liu, Kai-Qi Su.
